# Supplementary material for: A Core Outcome Set to evaluate the impact of prognostication in people living with advanced cancer: An international consensus study
Source: PLoS One. 2026 Apr 9;21(4):e0346683. doi: 10.1371/journal.pone.0346683 (PMC13065008; doi:10.1371/journal.pone.0346683)
Supplement: S1 File — (PDF) [file pone.0346683.s001.pdf]

Comparison of outcome identified from the systematic review and interviews

Outcome domains	Outcomes<br>(*n* = 86)	Systematic review<br>(*n* = 70)	Interviews<br>(*n* = 33)
Mortality/survival	Length of survival	X	X
Psychiatric outcomes	Depression	X	X
	Anxiety	X	X
	Psychological/mental status	X	
	Psychological distress	X	
	Post-traumatic stress disorder	X	
	Panic disorder	X	
Physical outcomes	Pain	X	X
	Drowsiness	X	
	Nausea	X	
	General malaise	X	
	Weakness	X	
	Breathlessness	X	
Spiritual/religious/existential functioning/wellbeing	Hopefulness/maintaining hope	X	X
	Preparedness for end-of-life	X	
	Hopelessness/loss of hope	X	X
	Desire for death	X	X

	Being at peace with dying	X	
	Perceived sense of burden on others	X	
	Dissatisfaction with life	X	
	Sense of control		X
	Loss of control	X	
	Loss of dignity	X	
	Loss of interest/pleasure	X	
	Loss of resilience	X	
	Wish to live	X	X
	Worry about dying	X	
	Sense of suffering	X	
	Spiritual and religious coping		X
	Spiritual crisis	X	
Emotional functioning/wellbeing	Disbelief, shock, and denial		X
	Avoidance of prognosis		X
	Avoidance/denial	X	
	Fixation on prognosis		X
	Mental/emotional preparation for end-of-life		X
	Achieving/prioritising personal goals and values		X

	Use of coping strategies/mechanisms	X	
	Having the opportunity to say goodbye to loved ones	X	X
	Caregiver regret	X	
	Emotional distress	X	X
	Frustration	X	
	Prognostic acceptance	X	X
	Bereavement in caregivers	X	
	Anticipatory grief		X
	Pre-loss grief in caregivers	X	
Cognitive functioning	Cognitive function	X	
Social functioning	Quality of relationships with others		X
	Quality of communication between patient and family/friends		X
	Communication between patient and family/friends	X	
	Patient-caregiver relationship	X	
	Social isolation	X	
Global quality of life	Quality of life	X	
Delivery of care	Treatment preferences (including life-sustaining treatments)	X	
	Shared decision making		X
	End-of-life/advance care planning		X

	Do-not-resuscitate order completion	X	
	Conflicting preferences for prognostic information between patients and caregivers	X	X
	Having a survival timeframe	X	
	Needing additional information	X	
	End-of-life planning/discussions	X	
	Change in information needs/preferences	X	
	Dying in hospital	X	
	Dying in a preferred location	X	X
	Quality of death	X	
	Preference for hospice care	X	
	Preference for comfort care	X	
	Patient-doctor relationship	X	X
	Family present at time of death	X	
	Advance directives in place	X	
	Participation in clinical trials	X	
	Decisional satisfaction	X	
	Place of care		X
	Access to practical support		X
	Access to financial support		X

	Family informed about imminent death	X	
	Bereavement support offered to family	X	
Perceived health status	Prognostic awareness	X	
	Prognostic understanding	X	X
	Being aware of prognostic uncertainty	X	X
Personal circumstances	Getting affairs in order	X	X
	Living will or durable power of attorney in place	X	
	Financial concerns	X	
Hospital/hospice use	Hospice enrolment	X	
	Length of hospital admission	X	
	Admission to hospital (rate per week)	X	
Caregiver/family burden	Caregiver/family burden		X
