# Supplementary material for: A Core Outcome Set to evaluate the impact of prognostication in people living with advanced cancer: An international consensus study
Source: PLoS One. 2026 Apr 9;21(4):e0346683. doi: 10.1371/journal.pone.0346683 (PMC13065008; doi:10.1371/journal.pone.0346683)
Supplement: S2 File — (PDF) [file pone.0346683.s002.pdf]

Rating of outcomes by stakeholder group in round 1 of the Delphi survey

Outcomes	Patients (*n* = 10)				Informal caregivers (*n* = 11)				Clinicians (*n* = 18)				Academics/researchers (*n* = 10)				Overall (*n* = 49)				Consensus
	1-3	4-6	7-9	UN	1-3	4-6	7-9	UN	1-3	4-6	7-9	UN	1-3	4-6	7-9	UN	1-3	4-6	7-9	UN	
	%	%	%	%	%	%	%	%	%	%	%	%	%	%	%	%	%	%	%	%	
Length of survival	0	10	80	10	18	9	64	9	0	17	83	0	10	30	60	0	6	16	73	4	
Pain	0	10	90	0	0	18	73	9	6	28	61	6	10	50	40	0	4	27	65	4	
Drowsiness	0	40	60	0	9	27	64	0	6	50	39	6	10	40	50	0	6	41	51	2	
Nausea	0	20	70	0	9	27	64	0	11	50	22	17	10	40	50	0	8	39	47	6	
General malaise	0	40	50	0	9	36	55	0	0	44	50	6	10	40	50	0	4	43	51	2	
Weakness	0	50	50	0	9	27	64	0	0	44	50	6	10	40	50	0	4	41	53	2	
Breathlessness	0	40	60	0	9	36	55	0	0	44	44	11	10	10	70	10	4	35	55	6	
Depression	10	20	70	0	9	18	73	0	6	39	56	0	10	20	70	0	8	27	65	0	
Anxiety	0	20	70	0	0	9	82	9	6	33	61	0	10	20	70	0	4	24	69	2	
Psychological/mental status	10	30	50	0	9	9	82	0	6	22	61	11	0	30	70	0	6	24	65	4	
Psychological distress	0	30	70	0	9	18	73	0	6	22	72	0	0	20	80	0	4	22	73	0	
Spectrum of hope	0	20	70	0	9	9	82	0	0	22	78	0	10	40	40	10	6	22	69	2	
Being at peace with dying	0	30	70	0	9	18	73	0	0	28	72	0	10	40	40	10	4	29	65	2	
Spiritual and religious coping	10	30	40	10	18	9	73	0	0	44	50	6	20	50	30	0	12	35	49	4	
Spiritual crisis	20	50	20	0	18	27	55	0	6	33	56	6	0	50	50	0	12	39	47	2	
Loss of interest/pleasure	0	30	70	0	18	36	45	0	0	33	61	6	0	30	70	0	4	33	61	2	
Loss of resilience	0	40	60	0	9	27	64	0	0	33	61	6	10	40	50	0	4	35	59	2	
Loss of dignity	0	20	80	0	9	27	64	0	0	33	67	0	10	20	50	20	4	27	65	4	
Dissatisfaction with life	10	50	30	0	18	27	55	0	0	28	67	6	10	30	50	10	10	33	53	4	
Perceived sense of burden on others	0	10	90	0	9	27	64	0	0	28	72	0	10	20	60	10	4	22	71	2	
Sense of suffering	0	30	60	10	9	18	73	0	0	28	67	6	0	20	80	0	2	24	69	4	
Sense of control	0	30	70	0	18	0	82	0	0	33	67	0	10	40	50	0	6	27	67	0	
Desire for death	10	20	70	0	27	0	64	9	0	28	72	0	0	50	50	0	8	24	65	2	

Wish to live	0	10	80	0	9	0	82	9	6	39	56	0	10	50	40	0	8	27	63	2
Worry about dying	10	10	70	0	18	0	82	0	0	28	72	0	0	20	80	0	6	18	76	0
Disbelief, shock, and denial	0	0	80	10	9	27	55	9	0	44	56	0	20	20	50	10	8	27	59	6
Avoidance of prognosis	10	10	70	0	18	36	36	9	6	44	50	0	20	30	50	0	14	33	51	2
Prognostic acceptance	10	10	70	0	18	27	55	0	0	39	56	6	10	20	70	0	10	27	61	2
Emotional distress	0	20	70	0	18	18	64	0	6	28	61	6	10	10	80	0	10	20	67	2
Use of coping strategies/mechanisms	0	10	80	0	27	9	55	9	0	33	67	0	10	20	70	0	8	22	67	2
Fixation on prognosis	0	30	60	0	27	27	45	0	17	33	50	0	10	40	50	0	16	33	51	0
Mental/emotional preparation for end-of-life	0	10	80	0	18	9	64	9	0	33	67	0	10	10	80	0	8	18	71	2
Achieving/prioritising personal goals and values	0	40	40	10	18	18	64	0	0	28	72	0	0	20	80	0	6	27	65	2
Anticipatory grief in patients	0	20	70	0	27	18	55	0	0	28	72	0	0	50	50	0	8	29	63	0
Anticipatory grief in informal caregivers	0	20	80	0	18	27	55	0	0	33	61	6	10	30	60	0	6	29	63	2
Having the opportunity to say goodbye to loved ones	10	10	80	0	27	0	73	0	0	33	67	0	10	10	80	0	10	16	73	0
Decisional satisfaction	0	0	90	0	18	9	64	9	0	50	50	0	10	20	70	0	6	27	65	2
Regret in informal caregivers	0	40	50	0	18	18	55	9	0	67	33	0	10	40	50	0	6	47	45	2
Bereavement in informal caregivers	0	20	60	10	9	18	73	0	6	50	44	0	0	30	70	0	4	35	59	2
Cognitive function	0	10	80	0	9	9	82	0	11	28	61	0	10	40	50	0	8	24	67	0
Quality of communication between patient and family/friends	0	20	80	0	9	9	82	0	11	33	50	6	0	30	70	0	6	24	67	2
Quality of patient-informal caregiver relationship	0	0	100	0	9	9	82	0	6	44	50	0	0	30	70	0	4	24	71	0
Quality of relationships with others	0	30	70	0	9	9	82	0	11	50	39	0	10	50	40	0	8	37	55	0
Social isolation	0	40	60	0	18	9	73	0	11	33	56	0	0	50	50	0	8	33	59	0
Quality of life	0	10	90	0	9	18	73	0	0	22	78	0	10	20	70	0	4	18	78	0
Treatment/care preferences	0	0	90	10	9	9	82	0	0	6	94	0	10	30	60	0	4	10	84	2
Shared decision making	0	10	90	0	9	9	82	0	0	17	83	0	0	10	90	0	2	12	86	0
End-of-life/advance care planning	0	20	80	0	9	9	82	0	0	17	78	6	20	10	70	0	6	14	78	2
Information needs/preferences	0	0	100	0	9	9	82	0	0	22	78	0	0	10	90	0	2	12	86	0
Patient-doctor relationship	0	10	90	0	9	0	91	0	0	33	61	6	0	20	80	0	2	18	78	2

Family informed about imminent death	10	20	70	0	9	9	82	0	0	22	78	0	10	40	40	10	6	22	69	2
Family present at time of death	10	40	50	0	9	0	91	0	6	44	50	0	0	50	50	0	6	35	59	0
Place of care	0	10	90	0	9	0	91	0	6	33	61	0	10	30	60	0	6	20	73	0
Place of death	10	10	80	0	9	0	91	0	6	39	56	0	10	30	60	0	8	22	69	0
Quality of death	10	10	80	0	9	0	91	0	0	28	72	0	10	10	70	10	6	14	78	2
Access to practical support	0	0	100	0	18	0	82	0	0	33	67	0	0	60	40	0	4	24	71	0
Access to financial support	0	0	80	10	27	9	64	0	11	33	56	0	10	40	50	0	12	22	61	4
Participation in clinical trials/research	10	20	70	0	18	27	55	0	17	67	17	0	20	40	30	10	16	43	39	2
Prognostic awareness	10	0	80	10	9	0	91	0	0	17	83	0	0	30	70	0	4	12	82	2
Prognostic understanding	10	0	90	0	9	9	82	0	0	17	83	0	0	30	70	0	4	14	82	0
Being aware of prognostic uncertainty	0	10	90	0	9	18	73	0	0	22	78	0	0	20	80	0	2	18	80	0
Practical/logistical preparation for end-of-life	10	20	70	0	9	9	82	0	0	17	83	0	0	40	60	0	4	20	76	0
Financial concerns	0	10	70	10	18	0	82	0	11	33	56	0	10	30	60	0	10	20	65	4
Hospice enrolment	0	0	90	10	9	0	91	0	11	33	56	0	0	60	40	0	6	24	67	2
Admission to hospital	0	0	80	10	9	0	73	18	11	39	50	0	10	50	40	0	8	27	59	6
Length of hospital admission	0	10	80	10	9	9	82	0	11	33		0	0	40	50	10	6	24	65	4
Informal caregiver/family challenges	0	20	70	0	18	9	73	0	6	33	61	0	0	60	40	0	6	31	61	2

Outcomes that reached ‘consensus in’ (≥70% of all participants rated the outcome as ‘critical’ importance (7-9) AND ≤15% as ‘low’ importance (1-3))

Outcomes that did not reach consensus

Outcomes that reached consensus for inclusion in each stakeholder group

UN: Unable to rate
