# Supplementary material for: A Core Outcome Set to evaluate the impact of prognostication in people living with advanced cancer: An international consensus study
Source: PLoS One. 2026 Apr 9;21(4):e0346683. doi: 10.1371/journal.pone.0346683 (PMC13065008; doi:10.1371/journal.pone.0346683)
Supplement: S3 File — (PDF) [file pone.0346683.s003.pdf]

Rating of outcomes by stakeholder group in round 2 of the Delphi survey

Outcomes	Patients (*n* = 4)				Informal caregivers (*n* = 5)				Clinicians (*n* = 15)				Academics/researchers (*n* = 7)				Overall (*n* = 31)				Consensus
	1-3	4-6	7-9	UN	1-3	4-6	7-9	UN	1-3	4-6	7-9	UN	1-3	4-6	7-9	UN	1-3	4-6	7-9	UN	
	%	%	%	%	%	%	%	%	%	%	%	%	%	%	%	%	%	%	%	%	
Length of survival	0	0	100	0	0	20	80	0	0	13	87	0	0	14	86	0	0	13	87	0	
Pain	0	0	100	0	0	20	80	0	0	20	80	0	0	43	57	0	0	23	77	0	
Drowsiness	0	100	0	0	20	40	40	0	7	40	53	0	0	43	57	0	6	48	45	0	
Nausea	0	50	50	0	0	0	100	0	7	53	40	0	0	71	29	0	3	48	48	0	
General malaise	0	50	50	0	0	20	80	0	7	40	53	0	0	57	43	0	3	42	55	0	
Weakness	0	25	75	0	0	40	60	0	7	33	60	0	0	43	57	0	3	35	61	0	
Breathlessness	0	50	50	0	0	40	60	0	7	40	53	0	0	43	57	0	3	42	55	0	
Physical functioning	0	0	100	0	0	40	60	0	0	7	93	0	0	43	57	0	0	19	81	0	
Depression	0	25	75	0	0	40	60	0	0	20	80	0	0	43	57	0	0	29	71	0	
Anxiety	0	25	75	0	20	20	60	0	0	20	80	0	0	57	43	0	3	29	68	0	
Psychological/mental status	0	0	100	0	20	20	60	0	0	13	87	0	0	29	71	0	3	16	81	0	
Psychological distress	0	25	75	0	20	20	60	0	0	13	87	0	0	14	86	0	3	16	81	0	
Spectrum of hope	0	0	100	0	0	40	60	0	0	13	87	0	0	43	57	0	0	23	77	0	
Being at peace with dying	0	0	100	0	20	0	40	40	0	13	87	0	0	29	71	0	3	13	77	6	
Spiritual and religious coping	0	50	50	0	40	40	0	20	0	67	33	0	0	71	29	0	6	61	29	3	
Spiritual/religious/existential crisis	25	50	25	0	60	20	20	0	0	20	80	0	0	71	29	0	13	35	52	0	
Loss of interest/pleasure	0	50	50	0	40	20	40	0	0	33	67	0	0	43	57	0	6	35	58	0	
Loss of resilience	0	0	100	0	20	40	40	0	0	27	73	0	0	43	57	0	3	29	68	0	
Loss of dignity	0	0	100	0	20	0	80	0	0	7	93	0	0	29	71	0	3	10	87	0	
Dissatisfaction with life	0	75	25	0	20	20	40	20	0	40	60	0	0	57	43	0	3	45	48	3	
Perceived sense of burden on others	0	0	100	0	40	20	40	0	0	20	80	0	0	43	57	0	6	23	71	0	
Sense of suffering	0	0	100	0	20	20	60	0	0	13	87	0	0	29	71	0	3	16	81	0	
Sense of control	0	0	100	0	20	0	80	0	0	20	80	0	0	29	71	0	3	16	81	0	

Desire for death	0	50	50	0	20	20	20	40	0	20	80	0	0	29	71	0	3	26	65	6	
Wish to live	0	25	75	0	20	0	20	60	0	40	60	0	0	43	57	0	3	32	55	10	
Worry about dying	0	0	100	0	40	0	60	0	0	20	80	0	0	14	86	0	6	13	81	0	
Disbelief, shock, and denial	0	50	50	0	20	20	60	0	0	53	47	0	0	86	14	0	3	55	42	0	
Avoidance of prognosis	0	75	25	0	40	40	20	0	0	67	33	0	0	57	43	0	6	61	32	0	
Prognostic acceptance	0	25	75	0	20	20	40	20	0	60	40	0	0	43	57	0	3	45	48	3	
Emotional distress	0	0	100	0	40	0	60	0	0	13	87	0	0	43	57	0	6	16	77	0	
Use of coping strategies/mechanisms	0	0	100	0	40	0	60	0	0	33	67	0	0	43	57	0	6	26	68	0	
Fixation on prognosis	0	75	25	0	40	40	0	20	0	53	47	0	0	71	29	0	6	58	32	3	
Mental/emotional preparation for end-of-life	0	0	100	0	20	0	60	20	0	33	67	0	0	29	71	0	3	23	71	3	
Achieving/prioritising personal goals and values	0	25	75	0	20	40	40	0	0	47	53	0	0	14	86	0	3	35	61	0	
Anticipatory grief in patients	0	50	50	0	40	0	60	0	0	40	60	0	0	57	43	0	6	39	55	0	
Anticipatory grief in informal caregivers	0	0	100	0	20	40	40	0	0	40	60	0	0	43	57	0	3	35	61	0	
Having the opportunity to say goodbye to loved ones	0	0	100	0	20	20	60	0	0	13	87	0	0	14	86	0	3	13	84	0	
Decisional satisfaction	0	50	50	0	40	20	40	0	0	20	80	0	0	43	57	0	6	29	65	0	
Regret in informal caregivers	0	75	25	0	20	20	60	0	0	67	33	0	0	29	71	0	3	52	45	0	
Bereavement in informal caregivers	0	0	75	25	20	20	60	0	0	47	53	0	0	14	86	0	3	29	65	3	
Cognitive function	0	25	75	0	20	0	80	0	0	33	67	0	0	57	43	0	3	32	65	0	
Quality of communication between patient and family/friends	0	0	100	0	20	0	80	0	0	27	73	0	0	14	86	0	3	16	81	0	
Quality of patient-informal caregiver relationship	0	0	100	0	20	0	80	0	7	27	67	0	0	14	86	0	6	16	77	0	
Quality of relationships with others	0	50	50	0	20	40	40	0	7	53	40	0	0	57	43	0	6	52	42	0	
Social isolation	0	0	100	0	20	40	40	0	7	27	67	0	0	29	71	0	6	26	68	0	
Quality of life	0	0	100	0	0	20	80	0	0	27	73	0	0	29	71	0	0	23	77	0	
Treatment/care preferences	0	0	100	0	20	0	80	0	0	7	93	0	0	0	100	0	3	3	94	0	
Shared decision-making	0	0	100	0	20	0	80	0	0	20	80	0	0	0	100	0	3	10	87	0	
End-of-life/advance care planning	0	0	100	0	20	0	80	0	0	7	93	0	0	29	71	0	3	10	87	0	
Information needs/preferences	0	0	100	0	20	0	80	0	0	20	80	0	0	0	100	0	3	10	87	0	

Patient-clinician relationship	0	0	100	0	20	0	80	0	0	7	93	0	0	14	86	0	3	6	90	0
Family informed about imminent death	0	0	100	0	20	0	80	0	0	7	93	0	0	14	86	0	3	6	90	0
Family present at time of death	0	25	75	0	20	0	80	0	7	27	67	0	0	14	86	0	6	19	74	0
Place of care	0	0	100	0	20	0	80	0	0	47	53	0	0	14	86	0	3	26	71	0
Place of death	0	25	75	0	20	0	80	0	0	40	60	0	0	14	86	0	3	26	71	0
Quality of death	0	0	100	0	20	0	80	0	0	27	73	0	0	0	100	0	3	13	84	0
Access to practical support	0	0	100	0	0	20	80	0	0	20	80	0	0	29	71	0	0	19	81	0
Access to financial support	0	25	75	0	20	20	60	0	0	47	53	0	0	43	57	0	3	39	58	0
Participation in clinical trials/research	0	50	50	0	20	20	60	0	7	67	27	0	0	71	29	0	6	58	35	0
Prognostic awareness	0	0	100	0	20	0	80	0	0	13	87	0	0	14	86	0	3	10	87	0
Prognostic understanding	0	0	100	0	20	0	80	0	0	13	87	0	0	14	86	0	3	10	87	0
Being aware of prognostic uncertainty	0	0	100	0	20	20	60	0	0	20	80	0	0	14	86	0	3	16	81	0
Practical/logistical preparation for end-of-life	0	0	100	0	20	0	80	0	0	0	100	0	0	29	71	0	3	6	90	0
Financial concerns	0	50	50	0	40	0	60	0	7	47	47	0	0	29	71	0	10	35	55	0
Hospice enrolment	0	75	25	0	20	20	60	0	0	40	60	0	0	29	71	0	3	39	58	0
Admission to hospital	0	75	25	0	20	20	60	0	0	40	60	0	0	29	71	0	3	39	58	0
Length of hospital admission	0	75	25	0	20	20	60	0	0	27	73	0	0	14	86	0	3	29	68	0
Informal caregiver/family challenges	0	0	100	0	20	20	60	0	0	53	47	0	0	43	57	0	3	39	58	0

Outcomes that reached ‘consensus in’ (≥70% of all participants rated the outcome as ‘critical’ importance (7-9) AND ≤15% as ‘low’ importance (1-3))

Outcomes that did not reach consensus

Outcomes that reached consensus for inclusion in each stakeholder group

UN: Unable to rate
