# Supplementary material for: A Core Outcome Set to evaluate the impact of prognostication in people living with advanced cancer: An international consensus study
Source: PLoS One. 2026 Apr 9;21(4):e0346683. doi: 10.1371/journal.pone.0346683 (PMC13065008; doi:10.1371/journal.pone.0346683)

Analysis of attrition bias in round 1 ratings in each stakeholder group

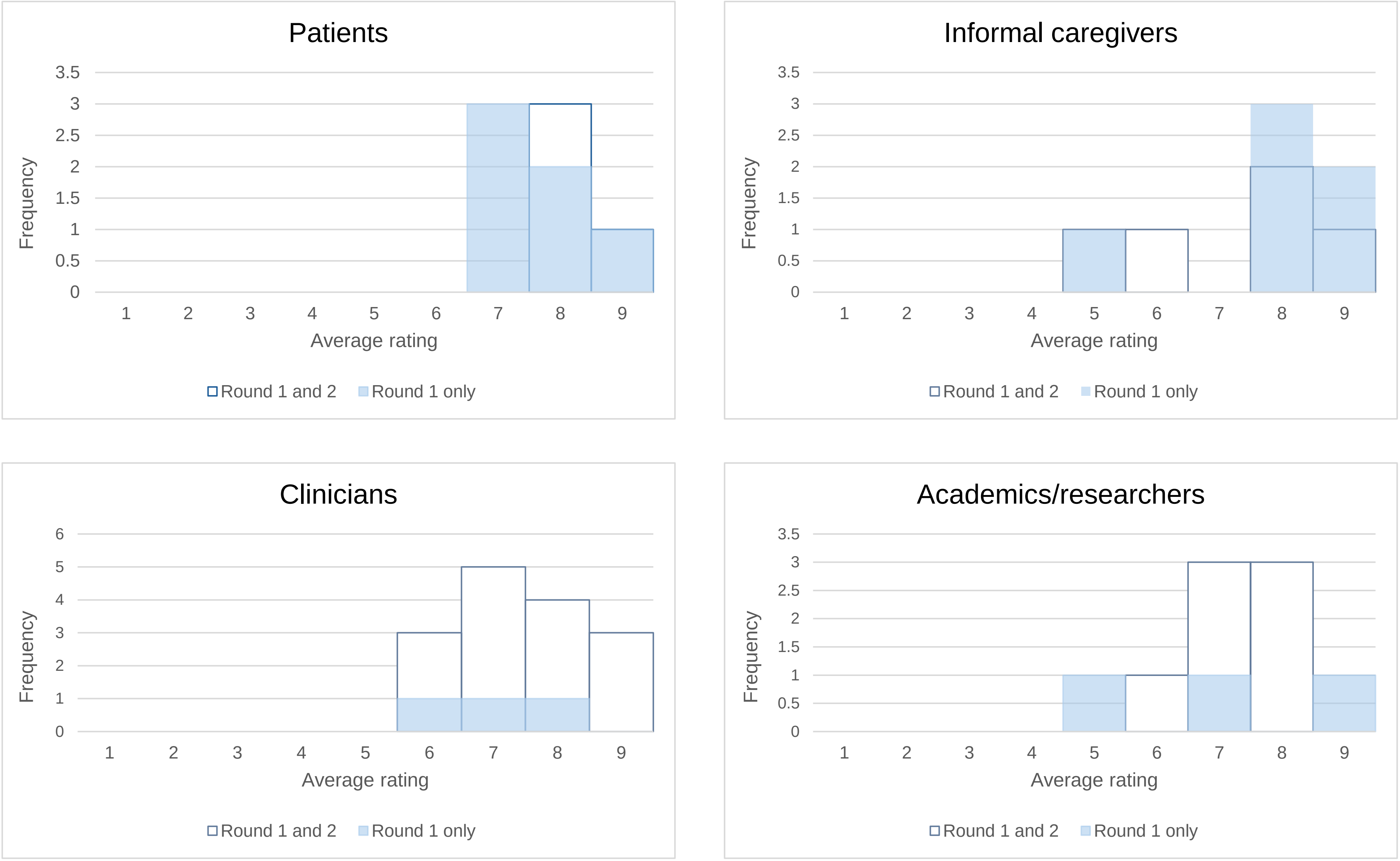

Analysis of attrition bias in round 1 ratings for all participants

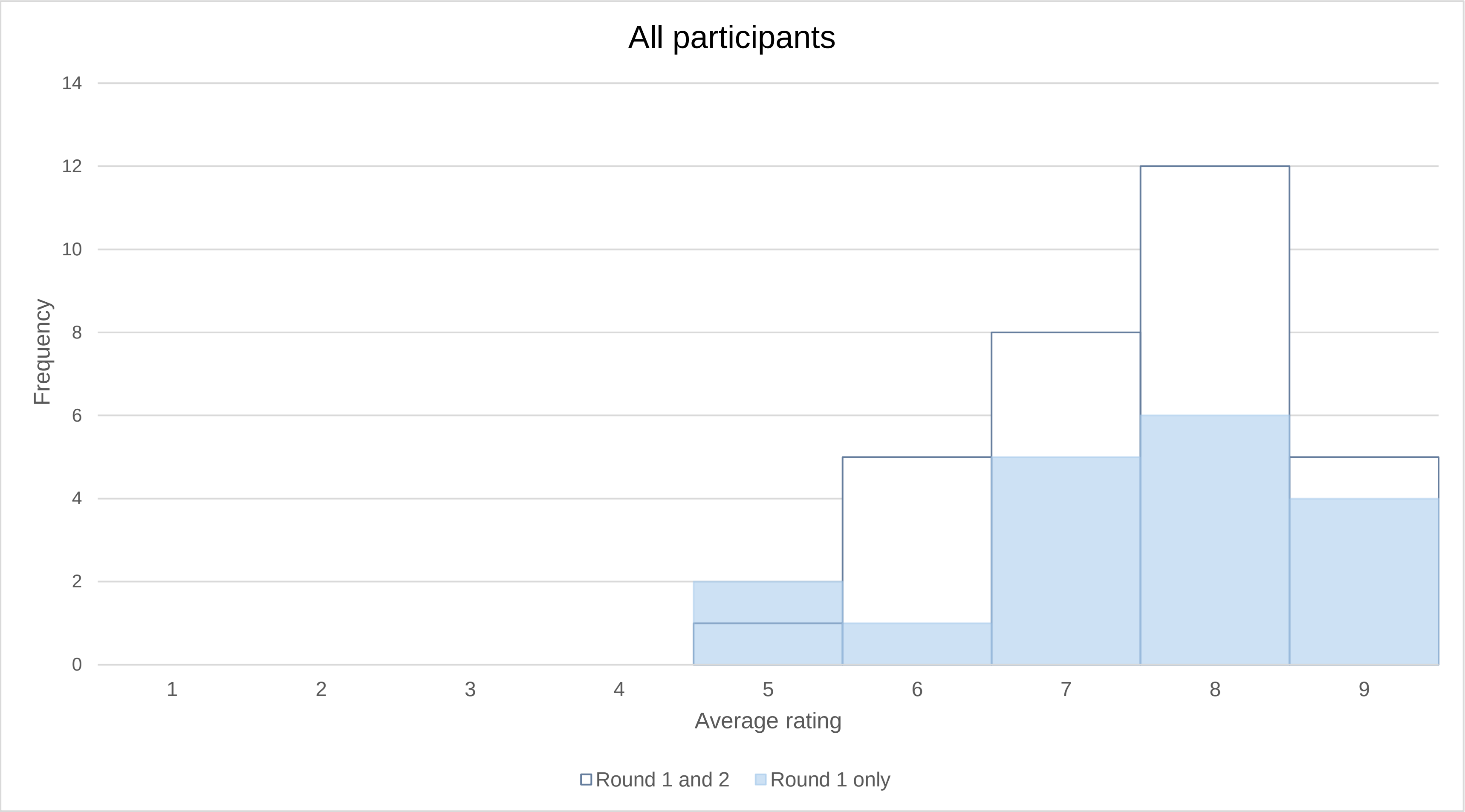

Supplement: S4 File — (PDF) [file pone.0346683.s004.pdf]
