# Supplementary material for: A Core Outcome Set to evaluate the impact of prognostication in people living with advanced cancer: An international consensus study
Source: PLoS One. 2026 Apr 9;21(4):e0346683. doi: 10.1371/journal.pone.0346683 (PMC13065008; doi:10.1371/journal.pone.0346683)
Supplement: S5 File — (PDF) [file pone.0346683.s005.pdf]

Rating of outcomes in the consensus meeting

Outcomes	1-3 (%)	4-6 (%)	7-9 (%)	Consensus
Length of survival	17	8	75	Consensus out
Pain	36	0	64	Consensus out
Physical functioning	8	17	75	Consensus in
Depression	45	9	45	Consensus out
Psychological/mental status	8	17	75	Consensus in
Psychological distress	25	17	58	Consensus out
Spectrum of hope	42	17	42	Consensus out
Being at peace with dying	50	25	25	Consensus out
Loss of dignity	33	17	50	Consensus out
Perceived sense of burden on others	17	8	75	Consensus out
Sense of suffering	42	17	42	Consensus out
Sense of control	33	17	50	Consensus out
Worry about dying	33	8	58	Consensus out
Emotional distress	42	8	50	Consensus out
Mental/emotional preparation for end-of-life	17	0	83	Consensus out
Having the opportunity to say goodbye to loved ones	33	0	67	Consensus out
Quality of communication between patient and family/friends	17	8	75	Consensus out
Quality of patient-informal caregiver relationship	33	8	58	Consensus out
Quality of life	8	8	83	Consensus in
Treatment/care preferences	8	0	92	Consensus in

Shared decision making	25	8	67	Consensus out
End-of-life/advance care planning	8	0	92	Consensus in
Information needs/preferences	64	8	36	Consensus out
Patient-clinician relationship	25	0	75	Consensus out
Family informed about imminent death	33	0	67	Consensus out
Family present at time of death	58	8	33	Consensus out
Place of care	8	0	92	Consensus in
Place of death	33	0	67	Consensus out
Quality of death	8	0	92	Consensus in
Access to practical support	33	0	58	Consensus out
Prognostic awareness	25	8	67	Consensus out
Prognostic understanding	8	0	92	Consensus in
Being aware of prognostic uncertainty	33	0	67	Consensus out
Practical/logistical preparation for end-of-life	8	8	83	Consensus in
